# LITE-1 Photoreceptor Mediates Light-Induced Reversal of Ivermectin Paralysis in *Caenorhabditis elegans*

**DOI:** 10.17912/micropub.biology.001983

**Published:** 2026-04-11

**Authors:** Magera Shaw, Mylissa Stover, Crystal Shults, Jacob. R Manjarrez

**Affiliations:** 1 University of Florida, Gainesville, FL, US; 2 Oklahoma State University Center for Health Sciences, Tulsa, OK, US; 3 Biochemistry and Microbiology, Oklahoma State University Center for Health Sciences, Tulsa, OK, US

## Abstract

Ivermectin (IVM), a widely used anthelmintic and chemotherapeutic agent in both human and veterinary medicine, targets glutamate-gated chloride channels to induce paralysis in nematodes such as&nbsp; *Caenorhabditis elegans* . Traditionally, IVM-induced paralysis is assessed under brightfield microscopy. Here, we report that exposure to UV or blue wavelengths can induce spontaneous arousal from the IVM-paralyzed state, initiating with twitching and progressing to full swimming motion during light stimulation. This light-induced arousal response is absent in&nbsp; *lite-1* &nbsp;null mutants, implicating LITE-1 photoreceptors in mediating this effect. &nbsp;&nbsp;&nbsp;&nbsp;&nbsp;&nbsp;&nbsp;&nbsp;&nbsp;&nbsp;&nbsp;&nbsp;&nbsp;&nbsp;&nbsp;&nbsp;&nbsp;&nbsp;&nbsp;&nbsp;&nbsp;

**Figure 1. Light-induced arousal from ivermectin-induced paralysis:** (A–B) Ivermectin-paralyzed worms were exposed to 2 minutes of either UV (395/25 nm; Panel A) or blue (470/40 nm; Panel B) illumination. Each data point represents the latency (in seconds) to the first observed full-body wave for an individual worm. Worms that did not move during the 2-minute trial were assigned a value of 120 seconds. (C) Summary table of arousal responses across genotypes.&nbsp;&nbsp;

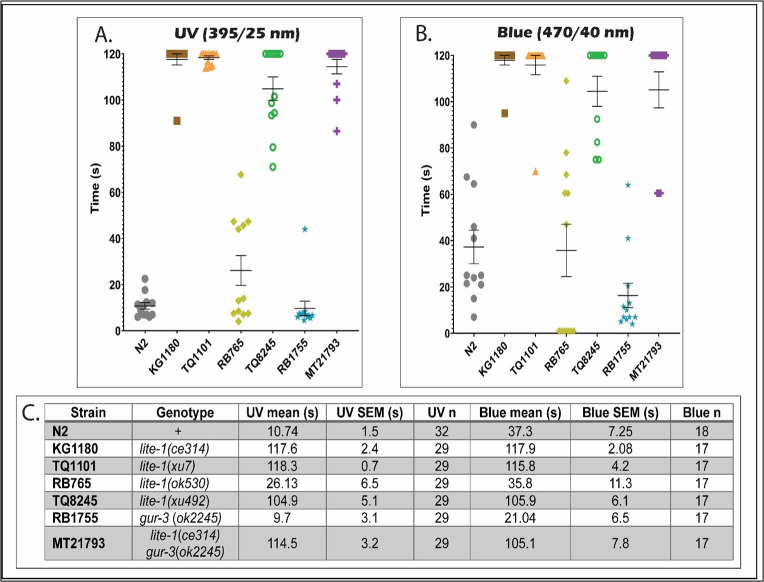



## Description

Ivermectin (IVM) is the first semi-synthetic macrocyclic lactone approved for veterinary use as a chemotherapeutic and broad-spectrum antiparasitic agent. It is known to kill&nbsp; *Caenorhabditis elegans* &nbsp;( *C. elegans* ) at therapeutic doses and has been widely used in paralytic assays at subtherapeutic concentrations. (Ardelli et al., 2009; Hibbs & Gouaux, 2011). IVM exerts its effects by binding to and activating glutamate-gated chloride channels in the muscles and nerves of nematodes and arthropods (Ardelli et al., 2009; Dent et al., 1997, 2000; Ghosh et al., 2012; Glendinning et al., 2011; Hibbs & Gouaux, 2011; Yates et al., 2003). The subtherapeutic paralytic state can be maintained over a wide range of concentrations and durations according to the previously published literature, presumably under standard brightfield imaging conditions (Ardelli et al., 2009; Castro et al., 2020; Chen & Kubo, 2018; Dent et al., 1997, 2000; Glendinning et al., 2011; Hernando & Bouzat, 2014; Le et al., 2019).

The photosensory-defective *lite-1* mutant has been described in several studies based on its failure to avoid UV, violet, and blue wavelengths (Edwards et al., 2008; Liu et al., 2010). However, this phenotype was initially identified by its ability to induce movement in otherwise paralyzed unc-31 mutants upon light stimulation (Ward et al., 2008). The nature of the underlying signal remains unidentified ; however, multiple studies are beginning to implicate neuropeptides in modulating this response, including NLP-10 *and* FLP-1 (Aoki et al., 2024; Dunkel et al., 2025).

Due to its insensitivity to blue light,&nbsp; *lite-1* &nbsp;is now routinely used to minimize background interference in GCaMP-based neural imaging (Ji, Madan, et al., 2021; Ji, Venkatachalam, et al., 2021; Kumar et al., 2023; Li et al., 2023; Toyoshima et al., 2020). A second light-sensitive gene,&nbsp; *gur-3 ,* has been associated with avoidance of extremely bright light (Bhatla & Horvitz, 2015). Although its role in light sensation is less well characterized,&nbsp; *gur-3* &nbsp;was included in this study to assess its potential involvement in the reversal of IVM-induced paralysis (Bhatla & Horvitz, 2015; Fang-Yen et al., 2015; Liu et al., 2010).&nbsp;

UV and blue spectrum spontaneous arousal after ivermectin treatment: We initially observed that IVM-induced paralysis in wild-type animals could be reversed by exposure to UV or blue light. To determine whether this spontaneous reversal was mediated by LITE-1 photoreceptors, we compared the responses of wild-type ( N2 ) animals, four different&nbsp; *lite-1* &nbsp;mutant alleles, a&nbsp; *gur-3 ( ok2245 )* &nbsp;mutant, and a&nbsp; *lite-1 ( ce314 ); gur-3 ( ok2245 )* &nbsp;double mutant. Upon illumination with UV or blue light, N2 ,&nbsp; *gur-3 ( ok2245 )* , and&nbsp; *lite-1 ( ok530 )* &nbsp;animals consistently exhibited an arousal response characterized by initial twitching followed by a return to full swimming motion during each light exposure. In contrast,&nbsp; *lite-1 ( ce314 )* ,&nbsp; *lite-1 ( xu7 )* ,&nbsp; *lite-1 ( xu492 )* , and the&nbsp; *lite-1 ( ce314 ); gur-3 ( ok2245 )* &nbsp;double mutant displayed minimal or no arousal events ( Figure 1A –C; Video S1). &nbsp;Green light (545/30 nm) did not trigger any movement following IVM-induced paralysis.

In contrast, the light-activated strains consistently returned to a paralyzed state once the UV or blue light stimulus was removed. However, the time required to resume paralysis varied, and we were unable to identify a consistent trend across trials.

The reduced penetrance shown for *lite-1 ( ok530 )* was confirmed not to be due to heterozygosity (Figure S1). However, because this strain was used without outcrossing, it remains possible that an additional mutation could compensate for the continued light-sensing function. Nonetheless, this study indicates that such a compensatory component is not associated with *gur-3* (Figure 1).

In summary, we demonstrate that UV and blue light stimulation can induce spontaneous arousal from IVM-induced paralysis in&nbsp; *C. elegans* , characterized by initial twitching followed by a return to full swimming motion. The reversal of paralysis triggered by light is strong in wild-type animals but is almost absent in severe *lite-1* photosensory mutants, suggesting that the LITE-1 protein plays a vital role in this response.&nbsp; This response may be mediated by neuropeptide signaling, which has been recently implicated in light-induced arousal pathways.

However, these results show the need to account for light-induced behavioral artifacts when designing experiments that use fluorophores excited by UV or blue wavelengths, especially in neuromuscular or behavioral assays, including ivermectin. While the exact molecular mechanisms remain unclear, our findings demonstrate that light stimulation can alleviate ivermectin-induced paralysis, even in strains with partially disrupted phototransduction pathways.

## Methods

Nematodes were cultured on OP50 , *E. coli* , and NGM media at 20°C. One-day-old adult *C. elegans* were picked from a non-starved culture and transferred onto an unseeded plate or liquid buffer containing the drug compound at 20°C for the duration of the experiment (Brenner, 1974).

A 25 mg/ml Ivermectin (Alfa Aesar) stock solution was diluted in 100% ethanol and stored at -20°C.

IVM-induced paralysis - One-day-old adults were individually transferred into a 96-well Black-Clear Bottom Plate (ThermoScientific 265301). Each well contained 10 µM IVM in M9(Castro et al., 2020). Plates were incubated at 20˚C for 30 minutes, which has been shown to induce paralysis, before prodding (Castro et al., 2020; Glendinning et al., 2011; Hernando & Bouzat, 2014), then UV and/or blue light stimulation.&nbsp; Each well contained a single worm that was stimulated with UV or blue light for 2 min, during which the movement was observed using a DMi8 Leica Thunder imager with a 10x objective exposed to 100% UV (395/30 nm) using a Spectra X light engine with a output of 295mW (395/25) or GFP (470/40nm) with a cyan output of 196mW (470/24). Movements were tallied when a worm made a full wave of its body (Lumencor, Inc., 2022).&nbsp;

PCR genotyping was performed using worm lysis buffer, freeze-heat lysis (freezing at -80 ^◦^ C then cycling at 60 ^◦^ C than at 95 ^◦^ C to inactivate the Proteinase K in the buffer), and using the lysate as a template for PCR with deletion-discriminating primers. PCR products were resolved on a 1.0% agarose E-Gel (Invitrogen) according to the manufacturer's instructions. Gels were imaged on an iBright Imaging Systems system, and banding patterns were scored as homozygous mutant (single *ok530* -sized band) or heterozygous (both WT- and *ok530* -sized bands).

## Reagents

Strain List:

**Table:** 

**Strain**	**Genotype**	**Source**
**N2**	Wild-type	Ken Miller
**KG1180**	*lite-1 ( ce314 ) *	Ken Miller
**TQ1101**	*lite-1 ( xu7 ) *	CGC
**TQ8245**	*lite-1 ( xu492 ) *	CGC
**RB765**	*lite-1 ( ok530 ) *	CGC
**RB1755**	*gur-3 ( ok2245 ) *	CGC
**MT21793**	*lite-1 ( ce314 ); gur-3 ( ok2245 ) *	CGC

&nbsp;

## Data Availability

Description: Genotyping for lite-1(ok530). Resource Type: Dataset. DOI: https://doi.org/10.22002/daj1g-d5k79
